# WNT-5A: signaling and functions in health and disease

**DOI:** 10.1007/s00018-015-2076-y

**Published:** 2015-10-29

**Authors:** Kuldeep Kumawat, Reinoud Gosens

**Affiliations:** 1 Department of Molecular Pharmacology, University of Groningen, Antonius Deusinglaan 1, 9713 AV Groningen, The Netherlands; 2 Groningen Research Institute for Asthma and COPD, University of Groningen, Groningen, The Netherlands

**Keywords:** Transcription, Receptors, Embryogenesis, Migration, Differentiation, Fibrosis, Cancer, Inflammation

## Abstract

WNT-5A plays critical roles in a myriad of processes from embryonic morphogenesis to the maintenance of post-natal homeostasis. WNT-5A knock-out mice fail to survive and present extensive structural malformations. WNT-5A predominantly activates β-catenin-independent WNT signaling cascade but can also activate β-catenin signaling to relay its diverse cellular effects such as cell polarity, migration, proliferation, cell survival, and immunomodulation. Moreover, aberrant WNT-5A signaling is associated with several human pathologies such as cancer, fibrosis, and inflammation. Thus, owing to its diverse functions, WNT-5A is a crucial signaling molecule currently under intense investigation with efforts to not only delineate its signaling mechanisms and functions in physiological and pathological conditions, but also to develop strategies for its therapeutic targeting.

## Introduction

WNT-5A is a member of the Wingless/integrase 1 (WNT) family of secreted glycoproteins. In humans, 19 WNT proteins (WNTs) are currently known that act as ligands for several membrane-bound receptors which includes 10 class Frizzled receptors (FZD), low-density lipoprotein receptor-related protein (LRP) 5/6 co-receptors, and many non-class FZD receptors, such as ROR1, ROR2, RYK, and PTK7 [1]. The intracellular WNT signaling is broadly classified into two main branches—β-catenin-dependent (canonical) and β-catenin-independent (non-canonical) WNT signaling. Due to the complexity and vast diversity of downstream signaling, the canonical and non-canonical nomenclature has become outdated. WNT/β-catenin signaling is initiated by binding of a WNT to a class FZD receptor and LRP5/6 co-receptors concluding a multimeric membrane signaling complex which results in the stabilization and cytosolic accumulation of transcriptional co-activator β-catenin. Ultimately, the stabilized β-catenin translocates to the nucleus where it associates with the T-cell factor/lymphoid enhancer-binding factor (TCF/LEF) transcription factors and activates WNT-target gene transcription [1]. In contrast, the β-catenin-independent signaling branches function independent of β-catenin and LRP5/6 and activate various signaling cascades involved in the regulation of cell polarity and movements, cytoskeletal reorganization, and gene transcription. Two of the best characterized β-catenin-independent WNT signaling pathways are the WNT/Ca^2+^ and WNT/planar cell polarity (PCP) pathways. The WNT/Ca^2+^ signaling pathway involves activation of Ca^2+^-dependent signaling molecules, including protein kinase C (PKC), Ca^2+^/calmodulin-dependent protein kinase II (CaMKII), and nuclear factor of activated T cell (NFAT), whereas the WNT/PCP pathway is mediated by RhoA signaling or activation of c-Jun N-terminal Kinases (JNKs) via small Rho-GTPases [2]. The WNT/Ca^2+^ pathway can also antagonize WNT/β-catenin signaling by phosphorylation of TCF/LEF transcription factors via activation of the TGF-β-activated kinase 1 (TAK1)-Nemo-like Kinase (NLK) cascade [3].

**Fig. 1:**
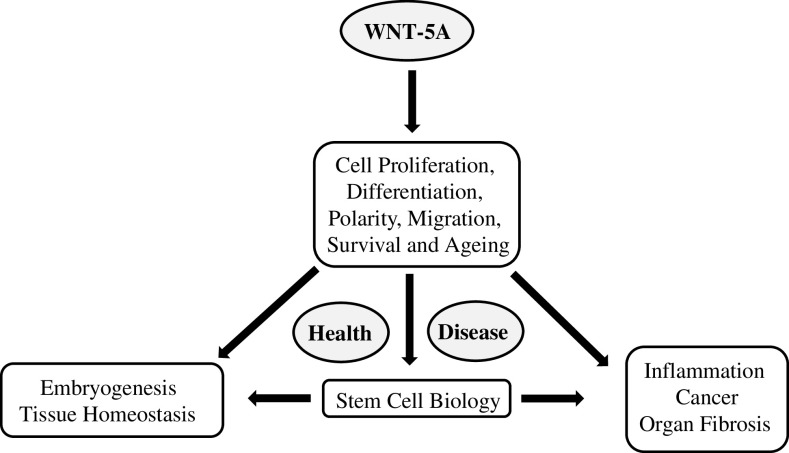
WNT-5A in health and disease. A schematic representation of key functions and pathologies associated with WNT-5A

WNT-5A, a prototypical WNT of β-catenin-independent branch, is highly conserved among species and plays key roles in the processes governing embryonic development, post-natal tissue homeostasis, and pathological disorders throughout the lifespan of an organism (Fig. 1) [4, 5]. Homozygous WNT-5A knock-out mice show perinatal lethality, primarily due to respiratory failure, and present extensive developmental abnormalities. It is involved in lung [6], heart [7], and mammary gland morphogenesis [8] and regulates stem cell renewal [9, 10], osteoblastogenesis [11, 12], and tissue regeneration [13]. In addition, aberrant WNT-5A expression and signaling is associated with various malignancies [14] and proinflammatory responses [15] as well as with lung [16], renal [17], and hepatic [18] fibrosis. WNT-5A signaling has also been implicated in ciliopathies [19] and WNT-5A antagonism counteracts vascular calcification [20]. We have recently reported increased WNT-5A expression in asthmatic airway smooth muscle cells [21] and have demonstrated that TGF-β induces WNT-5A expression in airway smooth muscle cells where it mediates expression of extracellular matrix proteins (ECM) [21] and participates in airway remodeling in asthma.

In view of the plethora of evidence associating WNT-5A with health and disease, there is considerable interest in understanding its biology. In this review, we discuss our current understanding of various aspects of WNT-5A signaling and its functions derived from studies in wide variety of in vivo models including *Drosophila*, *Xenopus,* and mouse; in vitro cell-based systems and patient-based reports.

## WNT-5A gene

WNT-5A cDNA was first isolated from mouse fetal tissue [22] followed by the isolation and sequencing from human cells [23]. The human WNT-5A gene is located on chromosome 3p14-p21. The WNT-5A gene generates two very identical transcripts by utilization of alternative transcription start sites and the corresponding upstream sequences are termed as promoter A and B [24] and their products as WNT-5A-L and WNT-5A-S, respectively [25]. Both the promoters have comparable transcriptional potential; their activity, however, is highly context dependent. WNT-5A promoter A has been suggested to be more active in human and murine fibroblasts as compared to promoter B [26]. Both the isoforms have similar biochemical properties such as stability, hydrophobicity, and signaling activity [25]. While the significance of individual WNT-5A isoforms is not completely understood, and it is not entirely clear whether they are functionally redundant, a recent study showed that they might have different functions [25]. When ectopically expressed, WNT-5A-L inhibited proliferation of various cancer cells lines, whereas WNT-5A-S leads to stimulation of growth [25].

## WNT-5A transcription

WNT-5A is a transcriptional target of an array of cytokines and growth factors. CUTL1 [27], STAT3 [28], TBX1 [29], and NFκB [30, 31] have been reported as transcription factors for WNT-5A in various cell types. We have recently shown that TGF-β induces expression of WNT-5A by engaging p38 and JNK signaling via TAK1 in airway smooth muscle cells [32]. This leads to the stabilization of β-catenin which then interacts with Sp1. Sp1, in turn, binds to the WNT-5A promoter and drives its expression [32]. TGF-β has also been shown to induce WNT-5A expression in mammary glands [8], primary fibroblasts [8], primary epithelial cells [8], and pancreatic cancer cells [27]. Similarly, proinflammatory factors such as interleukin (IL)-1β [31], tumor necrosis factor-α (TNF-α) [30], lipopolysaccharide (LPS)/interferon γ (IFNγ) [15], IL-6 family members-leukemia inhibitory factor (LIF) and cardiotrophin-1 (CT-1) [33], and high extracellular calcium concentration [34] all augment, whereas amino acid limitation [35] represses WNT-5A expression in various cell types. Collectively, this suggests that WNT-5A is a target of TGF-β and proinflammatory signaling which will be discussed below.

Interestingly, WNT-5A is also regulated at translational level via the numerous AU-rich motifs which are present in the evolutionary conserved 3′-untranslated region of mRNA [36]. AU-rich element binding proteins (ARE-binding proteins) associate with the AREs and tightly regulate their stability by posttranscriptional mechanisms. HuR, a member of embryonic lethal abnormal vision (ELAV) -like family of ARE-binding proteins, binds to the 3′-UTR AREs in WNT-5A mRNA and suppresses its translation [36].

## WNT-5A protein

**Fig. 2:**
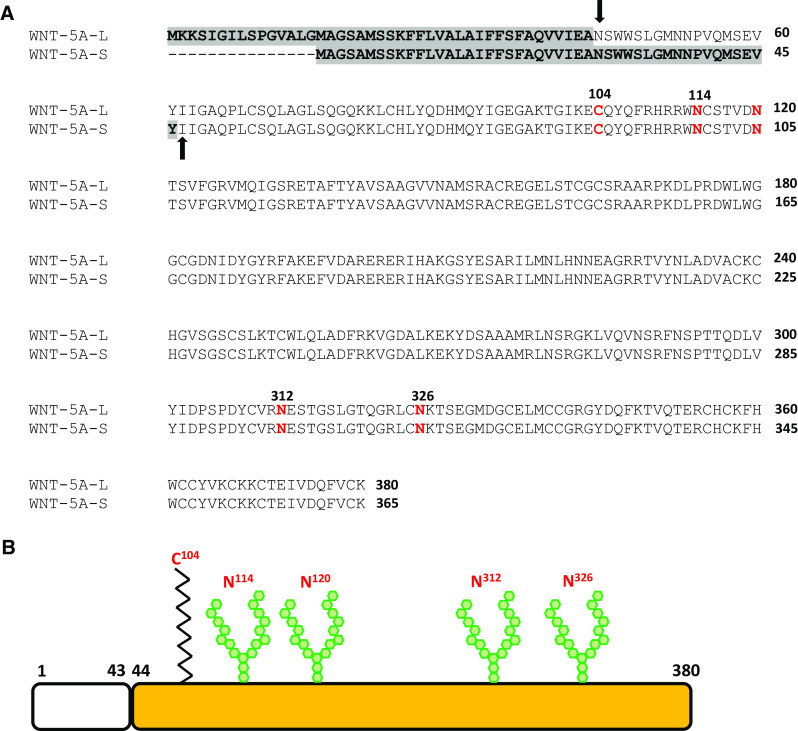
WNT-5A protein. **a** A comparative analysis of amino acid sequences of human WNT-5A-L and WNT-5A-S isoforms. *Gray* highlighted area represents N-terminal signal sequence in respective protein. *Bold arrows* mark the site of signal sequence cleavage and N-terminus of respective mature protein. The amino acids marked in *red-bold* represent posttranslational modification sites on protein backbone. *Number* represents the respective position of the amino acid from the first N-terminal amino acid. The protein sequences are taken from NCBI: NP_003383.2 (WNT-5A-L) and NP_001243034.1 (WNT-5A-S). **b** Diagrammatic representation of WNT-5A-L protein. N-terminal signal sequence is represented by *blank box*.  represents palmitoylation and  represents N-linked glycosylation on the protein backbone. The respective amino acids locations are marked above the modification sites. The N-linked glycosylation sites N312 and N326 correspond to N311 and N325 of mouse WNT-5A, respectively

WNT-5A-L and WNT-5A-S, composed of 380 and 365 amino acids, respectively, are heavily glycosylated and lipid-modified proteins. Each isoform consists of an N-terminal hydrophobic signal sequence, a conserved asparagine-linked oligosaccharide consensus sequence and about 22 highly conserved cysteine residues (Fig. 2a, b) [23]. Cleavage of the N-terminal signal sequence is predicted to generate mature protein containing either 343 or 338 amino acids [25]. However, N-terminal sequencing of mature WNT-5A isoforms revealed that WNT-5A-L is cleaved after the 43rd amino acid, whereas WNT-5A-S has much longer signal sequence with cleavage after the 46th amino acid, generating mature proteins containing 337 and 319 amino acids, respectively (Fig. 2a, b) [25]. Interestingly, mouse WNT-5A which is ~99 % homologous to human WNT-5A generates same mature protein as human WNT-5A-S [37]. In mouse WNT-5A, asparagine 114, 120, 311, and 325 have been identified as the N-linked glycosylation sites, whereas a palmitoylation has been identified at cysteine 104. The palmitoylation of WNT-5A is necessary for its binding to FZD_5_ and signaling activity but not required for its secretion [38, 39]. In contrast, glycosylation of WNT-5A is required for its secretion but dispensable for its signaling activity [38].

## WNT-5A: receptors and signaling

WNT-5A binding to receptor activates various β-catenin-independent signaling cascades; however, it can also activate WNT/β-catenin signaling depending on the cell- and receptor-context. WNT-5A can signal through multiple receptors and according to current understanding FZD_2_, FZD_3_, FZD_4_, FZD_5_, FZD_6_, FZD_7_, FZD_8_, RYK, ROR2, and CD146 may function as WNT-5A receptors [34, 37, 40–50].

WNT-5A has been shown to bind to FZD_2_ inducing intracellular calcium release and PKC activation in *Xenopus* [51] and zebrafish embryos [52] and WNT-5A-FZD_2_-induced calcium spikes in neurons are implicated in traumatic brain injury [53]. WNT-5A binds to FZD_2_ in a ROR1- or ROR2-dependent manner and recruits Disheveled (DVL) and β-arrestin to FZD_2_ leading to the clathrin-mediated internalization of FZD_2_ [40]. Internalization of FZD_2_ is essential for WNT-5A-induced Rac activation [40]. WNT-5A also induces clathrin-mediated internalization of FZD_4_ [54] in a PKC- and β-arrestin-dependent process and that of ROR2 in a PKC-dependent manner [47]. Similarly, binding of WNT-5A to FZD_5_ also leads to its internalization [38]. Internalization of receptors is considered as a critical step in WNT signaling and a reflection of active signaling. Although the exact mechanisms underlying the functional significance of receptor internalization are not clear, it is believed to facilitate intracellular signaling activation by recruitment of scaffolding proteins such as β-arrestin and may also facilitate the termination of signaling and receptor recycling [55].

We have recently demonstrated that WNT-5A signals through FZD_8_ and RYK receptors leading to the activation of Ca^2+^-NFATc1 and JNK signaling which mediates TGF-β-induced ECM expression in airway smooth muscle cells [21]. WNT-5A binding to FZD_7_ activates prosurvival PI3K/AKT cascade in human melanoma cells which can account for the resistance of these cells to BRAF inhibitors [48]. Similarly, WNT-5A can activate the PI3K/AKT cascade via FZD_3_ in human dermal fibroblasts and promotes integrin-mediated adhesion of these cells [41]. In contrast, WNT-5A-activated PI3K/AKT signaling induces migration in human osteosarcoma cells [56]. Similarly, WNT-5A induces migration in gastric cancer cells by activating PI3K/AKT pathway which phosphorylates and inactivates GSK-3β and activates RhoA leading to cytoskeleton remodeling [57]. Indeed, cytoskeletal reorganization and cell migration are major cellular effects of WNT-5A signaling.

WNT-5A is proposed to regulate cell fate via FZD_6_ in hair follicles [50], whereas it plays critical role in tuberculosis immunology via FZD_5_ regulating immune responses by antigen presenting cells and activated T cells in response to mycobacterium infection [42].

The FZDs belong to the class of seven transmembrane-spanning G protein-coupled receptors. Recent evidence shows a role for heterotrimeric G proteins in WNT-5A downstream signaling. For instance, G proteins are required for WNT-5A-induced JNK and NFκB activation in human neutrophils [58]. Similarly, WNT-5A activates G_αi/o_ proteins leading to Ca^2+^-dependent ERK1/2 activation in murine primary microglia [59] and HEK293 cells [60]. A recent study has shown that Daple (DVL-associating protein with a high frequency of leucine residues) functions as a non-receptor Guanine nucleotide exchange factor in WNT signaling which interacts and activates G_αi_ in response to WNT-5A stimulation [61]. This indicates that G protein coupling by FZDs is clearly a relevant physiological phenomenon, but whether coupling with heterotrimeric G proteins in FZD signaling is an absolute requirement or context-dependent remains unclear [62].

WNT-5A also binds to non-class FZD receptors including ROR2 and RYK receptor tyrosine kinases. ROR2 is a key receptor for WNT-5A-induced effects during development as demonstrated by remarkable phenotypic resemblance between the ROR2 and WNT-5A knock-out mice [63]. Multiple mechanisms have been suggested to explain the close functional relationship between WNT-5A and ROR2. WNT-5A interacts with ROR2 and VANGL2 to form a ternary complex leading to the casein kinase 1δ (CK1δ)-induced phosphorylation of VANGL2 which serves to relay the gradient effects of WNT-5A, thereby regulating WNT-5A-induced planar cell polarity and embryonic morphogenesis [64]. WNT-5A associates with FZD_7_ in the presence of ROR2 to form a complex required for DVL polymerization and activation of Rac-dependent WNT signaling [49]. WNT-5A activates ERK1/2 in intestinal epithelial cells via ROR2 [65], whereas it activates JNK-mediated c-Jun transcriptional activity to induce production of receptor activator of nuclear factor-κB (RANK), a regulator of osteoclast differentiation and activation, in osteoclast precursor cells via ROR2 [11]. WNT-5A engages ROR2 to activate JNK signaling and regulates cell movement [4, 66–68], whereas it induces assembly of DVL-atypical PKC (aPKC) and polarity complex (PAR3 and PAR6) to regulate neuronal differentiation and polarity [69, 70]. Thus, ROR2 participates in several key cellular functions of WNT-5A.

WNT-5A activates intracellular calcium release to fine tune neuronal growth by axonal outgrowth and repulsion. WNT-5A signals via RYK leading to calcium release from stores through IP_3_ receptors as well as calcium influx through transient receptor potential (TRP) channels inducing axonal outgrowth. On the other hand, simultaneous association of WNT-5A with RYK and FZD releases calcium from TRP channels without involvement of IP_3_ receptors and induces axonal repulsion [71]. WNT-5A also forms a ternary complex with RYK and VANGL2 to relay the WNT/PCP effects [72], whereas WNT-5A-RYK signaling is required for inhibition of reactive oxygen species (ROS) production and maintenance of hematopoietic stem cell quiescence [73].

Recently, WNT-5A binding to an adhesion molecule CD146 has also been described, leading to the recruitment of DVL2 to the complex and activation of downstream JNK signaling cascade [45]. CD146 has been linked to cell migration via RhoA-dependent cytoskeletal rearrangements [74]. In line with that, WNT-5A-CD146 axis regulates polarity and migration of cells [45, 75].

## Effects of WNT-5A on β-catenin signaling

Interestingly, in addition to activating the β-catenin-independent WNT pathway, WNT-5A can also have positive or negative regulatory effects on WNT/β-catenin signaling depending on the receptor- and cell-context. Indeed, a study has shown that WNT-5A can both activate and inhibit β-catenin-dependent WNT signaling during mouse embryonic development [76]. WNT-5A knock-out embryos show increased β-catenin activation in telencephalon and embryonic fibroblasts from WNT-5A knock-out animals show heightened response to WNT3A, a prototypical β-catenin-dependent signaling WNT [40]. Another study demonstrated that WNT-5A competes with WNT-3A for binding to FZD_2_, a receptor for both the WNTs, thereby inhibiting the WNT-3A-induced β-catenin signaling [40]. The WNT-5A-activated CaMKII–TAK1–NLK1 cascade has been implicated in WNT/β-catenin suppression [3]. In addition, WNT-5A inhibits WNT-3A-induced β-catenin signaling via ROR2 and CD146 [37, 45]. In hematopoietic stem cells, WNT-5A inhibits β-catenin signaling supposedly via suppression of ROS production [73]. Similarly, WNT-5A inhibits β-catenin signaling by promoting its degradation through an alternative E3 ubiquitin ligase complex composed of siah2-APC-Ebi [77]. Purified WNT-5A, on the other hand, can activate β-catenin-dependent transcription in the presence of FZD_4_ and LRP5 [37, 46]. Also, WNT-5A activates β-catenin signaling in pancreatic cancer cells [27, 78] and dermal fibroblasts [79]. Similarly, osteoblast-lineage cells from WNT-5A knock-out mice show reduced WNT/β-catenin signaling and WNT-5A pre-treatment potentiated the WNT/β-catenin signaling in bone marrow stromal cells via upregulation of LRP5 and LRP6 expression [80].

## Functions of WNT-5A

### Embryogenesis

WNT-5A has been identified for its key involvement in defining the body outgrowths in addition to many other specific features. WNT-5A expression is most abundant during early embryonic developmental stages between 10–14 days post conception [5, 22]. Importantly, homozygous WNT-5A knock-out mouse embryos show perinatal lethality underlining its vital role in embryogenesis. During development, regions undergoing extensive outgrowth like limbs, tail, and facial structures exhibit prominent WNT-5A expression where it is present in a graded fashion with the highest abundance at the tips of these structures and lowest in the proximal areas [5, 22]. WNT-5A knock-out leads to severe malformations in the outgrowth structures, a shortened anterior–posterior (A–P) and severely compromised proximal–distal (P–D) body axis. These malformations could be traced back to the underlying axial skeleton which exhibited a shortened vertebral column due to smaller vertebrae size and the absence of caudal vertebrae. The phenotype apparently originates from the critical role of WNT-5A as a mitogen required for the proliferation of the mesodermal progenitors early in embryonic development. The mesodermal stem cells which arise early in development can continue to develop in the primitive streak even in the absence of WNT-5A but lack the ability to divide and give rise to the progeny. Impaired self-renewal capacity leads to progressive depletion of the stock of these stem cells resulting in insufficient numbers of cells to develop the distal skeleton and leading to the absence of related structures [5].

Similar to WNT-5A knock-out mice, WNT-5A transgenic mice show perinatal lethality when WNT-5A is induced early in development exhibiting severe deformities resembling the WNT-5A knock-out phenotype [81]. Overexpression of WNT-5A induced malformations of limbs, tail, and facial structures. Underdeveloped limb skeletal elements, reduced number of tail vertebrae, and shortened upper and lower jaw bones constituted the mutant phenotype. Interestingly, overexpression of WNT-5A in later embryonic stages and in adult animals was well tolerated with no visible phenotype [81]. This study highlights a critical window during embryonic development when WNT-5A activity is most required [81].

Further studies have looked into the organ-specific developmental roles of WNT-5A and have identified a crucial role for distal morphogenesis of internal organs. For instance, WNT-5A knock-out mice fail to develop the genital tubercle [5] and have intestinal deformities [82]. Prominent WNT-5A expression is observed in the gut mesenchyme during intestinal morphogenesis which persists throughout the development of the small intestine [5, 83]. In line with that, WNT-5A knock-out mice show severe malformations in the small intestine with drastically reduced length and the presence of a secondary cavity. In addition, the mutants present an imperforated anus [82]. Interestingly, overexpression of WNT-5A during embryonic development also leads to gut malformations resembling the WNT-5A knock-out phenotype. Specifically, WNT-5A transgenic mice show shortening of the small and large intestine, caecum, and stomach and also present anal imperforation [81]. Of note, both the loss and overexpression of WNT-5A does not interfere with the intestinal differentiation or cell fate decisions. The underlying mechanisms that lead to the malformations observed in WNT-5A transgenic mice are not clear yet. However, the observation that overexpression of WNT-5A leads to the downregulation of ROR2 in intestine [81] could reveal the reason behind the similarities in both the WNT-5A transgenic and knock-out phenotypes. ROR2 is a receptor for WNT-5A and ROR2 knock-out mice show a phenotype resembling that of WNT-5A knock-out [63]. Therefore, increased expression of WNT-5A which leads to the downregulation of ROR2 could present a similar phenotype as ROR2 knock-out. Although the downstream WNT-5A signaling after overexpression remained intact, it is tempting to speculate that ROR2-dependent WNT-5A signaling is crucial for the embryonic development and that the loss of ROR2 in WNT-5A transgenic mice underlies the similarity with the WNT-5A knock-out phenotype.

Convergent extension (CE) is the critical morphogenetic movement during gastrulation wherein the germ layers narrow down mediolaterally resulting in the elongation of embryo from head to tail and shaping of body axis [84]. CE requires collective cell migration and cell intercalations. WNT-5A-activated signaling has been associated with CE movements [85–87] owing to its ability to regulate cell migration and polarity (as discussed in this review). Thus, embryonic structural abnormalities in WNT-5A knock-out and transgenic mice may not only arise from impaired proliferation but also due to derailed CE movements.

Lungs are complex organs with extensive branching, a large number of different types of specialized cells, and distinct P–D polarity. WNT-5A, as a major determinant of P–D polarity, is prominently expressed in the embryonic lungs [6, 22] where it is localized in both the mesenchymal and epithelial compartments. WNT-5A signaling is most enhanced at the tip and around the branching epithelium [6]. In later stages, WNT-5A is predominantly localized to the lung epithelium and attains a typical P–D gradient with most expression in the distal branching epithelium and almost no presence in the proximal regions [6]. Analysis of lungs obtained from WNT-5A knock-out mice revealed extensive developmental malformations. The trachea was truncated with reduced number of cartilages [6]. The branching morphogenesis of WNT-5A knock-out lungs was compromised as revealed by the increased number and overexpansion of terminal airways. Also, the intersaccular walls were thick and hypercellular indicating failed maturation of lungs in WNT-5A knock-out embryos. Further analysis revealed that loss of WNT-5A did not interfere with cell differentiation but led to hyperproliferation resulting in intersaccular septum thickening and disrupted vasculature [6]. Interestingly, WNT-5A knock-out lungs presented increased expression of sonic hedgehog/patched (SHH/PTC), fibroblast growth factor (FGF), and bone morphogenetic protein(BMP)-4 indicating the molecular mechanisms involved in the observed WNT-5A knock-out phenotype [6]. Notably, lungs of WNT-5A knock-out mice show resemblance with the FGF-10 knock-out [88], SHH knock-out [89, 90], SHH transgenic [91], and BMP-4 transgenic [92] lung phenotype, which underlines the interactive network of WNT-5A, FGF-10, SHH/PTC, and BMP-4 in lung development. Lung-specific WNT-5A transgenic expression also disrupts lung morphogenesis as demonstrated by dilated terminal airways, loss of branching, and smaller size of the lungs [93]. Interestingly, supporting a role for WNT-5A in regulating other signaling cascades, WNT-5A overexpression repressed SHH/PTC expression and distribution in the lung epithelium, whereas it augmented FGF-10 abundance in the mesenchyme [93]. While FGF-10 expression is increased, WNT-5A overexpression severely impairs the ability of epithelium to respond to FGF-10 [93]. Thus, WNT-5A fine-tunes the developmental signaling underlying the epithelial-mesenchyme communication which is required for proper lung morphogenesis [93].

WNT-5A expression is crucial for proper neuronal generation and axonal guidance during embryonic development and in post-natal life. WNT-5A knock-out mice show anomalies in the dopaminergic midbrain neuronal morphogenesis, organ innervation, and show increased neuronal apoptosis [94, 95]. Robust WNT-5A expression is detected in ventral midbrain where it promotes dopaminergic neurite and axonal growth [95]. In fact, WNT-5A promotes and cooperates with WNT/β-catenin signaling to generate midbrain dopaminergic neurons in vivo and in stem cells [39, 96], whereas WNT-5A expression in the sympathetic neurons is crucial for axonal branching for proper organ innervation via ROR1 and ROR2 receptors [63, 94]. WNT-5A can also signal via RYK to mediate cortical axonal growth and guidance [43, 71]. The absence of axonal guidance in both the ROR1/2- and RYK-deficient mice shows their function is non-redundant and the utilization of respective receptors may be context dependent.

WNT-5A is also required for proper cardiac morphogenesis as WNT-5A knock-out mice show severe defects in the septation of the cardiac outflow tract (OFT) [97]. OFT originates from an embryonic region called second heart field (SHF) which functions as a source of progenitor cells for development of most of the heart. WNT-5A is expressed in the pharyngeal mesoderm adjacent to cardiac neural crest cells in both mouse and chicken embryos and in the myocardial cell layer [97]. WNT-5A expression is induced in SHF by a transcription factor-TBX1 and loss of WNT-5A results in severe decline in the number of SHF progenitor cells and deployment of these progenitors to the OFT leading to cardiac deformities [7, 29, 98].

In summary, WNT-5A signaling is crucial to the development of internal organs and the formation of skeletal structures. Of importance, WNT-5A cooperates with other WNTs (e.g., WNT-11) and several non-WNT morphogens involved in development including TGF-β, BMPs, FGFs, and SHH signaling [8, 93, 99, 100]. This cooperation is essential, and while removing WNT-5A from this signaling network may lead to severe embryonic phenotypes, these phenotypes may not be attributed to WNT-5A alone. An intriguing example is the close cooperativity of WNT-5A and WNT-11 in the development of the second heart field in mice. Here, WNT-5A and WNT-11 are both required in suppressing WNT/β-catenin signaling in progenitors in the developing heart to allow for differentiation [7]. Recently, it was shown that WNT-5A and WNT-11 cooperate to regulate convergent extension movements leading to A–P axis formation in mice [101]. However, mice lacking WNT-5A (and not WNT-11) show severe A–P axis shortening and limb truncations highlighting a redundant role for WNT-11 in this process [5, 101, 102]. Clearly, WNT-5A is an essential component in the machinery that governs embryogenesis, and signaling by WNT-5A is non-redundant with that of other β-catenin-independent signaling WNTs.

### Migration

**Fig. 3:**
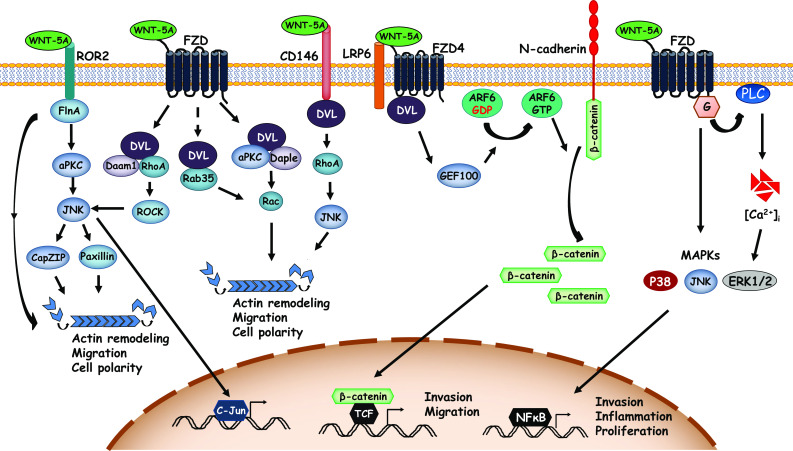
WNT-5A-activated signaling cascades in cell migration. Diagrammatic representation of few key signaling cascades engaged by WNT-5A to regulate actin cytoskeletal remodeling and cell migration. *ARF6* ADP-ribosylation factor 6, *GEF100* ARF-guanine nucleotide exchange protein 100, *FlnA* filamin A, *aPKC* atypical protein kinase C, *JNK* c-Jun N-terminal protein kinase, *CapZIP* CapZ-interacting protein, *DVL* disheveled, *Daam1* DVL-associated activator of morphogenesis 1, *Daple* DVL-associating protein with a high frequency of leucine residues, *ROCK* rho-associated kinase, *LRP6* low-density lipoprotein receptor-related protein 6, *G* G proteins, [*Ca* ^*2+*^]_*i*_ intracellular calcium release

Cell migration requires acquisition of new asymmetry and polarity along with reorganization of the cytoskeleton and breaking and/or reprocessing cell–cell and cell–substrate adhesions. As such, the WNT/PCP and WNT/Ca^2+^ pathways have been linked with migration of cells. Several studies have elucidated the significance and molecular mechanisms of WNT-5A-induced cell migration (Fig. 3). For instance, a study has identified the WNT-5A-ROR2 axis in regulating cell motility. WNT-5A interacts with ROR2 and induces its association with Filamin A, an actin binding protein, which, in turn, leads to formation of filopodia [103]. Filopodia are actin-based structures projecting at the leading edge of migrating cells and are important in formation of focal adhesions attaching to the substrate and facilitating directional cell movement [104]. WNT-5A-induced ROR2-Filamin A association activates aPKC which in turn activates JNK. Activated JNK may mediate cell migration by microtubule organizing center (MTOC) reorientation and actin remodeling via phosphorylation and activation of CapZ-interacting protein (CapZIP) [105]. In addition, JNK can also phosphorylate paxillin regulating focal adhesion complexes [106, 107] and modulating cell motility in response to WNT-5A. In another mechanism, WNT-5A induces cell migration via Daple-mediated Rac activation [108]. Daple interacts with DVL in response to WNT-5A and facilitates its interaction with aPKC consequently inducing Rac activation. This leads to cytoskeletal reorganization promoting lamellipodia formation and cell migration [108]. In addition to aPKC, WNT-5A can also employ Rab35 to activate Rac in a DVL-dependent manner and induce cell migration [109].

The WNT-5A-RhoA axis has been prominently linked with cytoskeletal remodeling and cell motility in various cell systems. WNT-5A induces RhoA activation via DVL and Daam1 in breast cancer cells [110] or via PI3K/AKT signaling in gastric cancer cells [57]. Activated RhoA, in turn, may engage other downstream pathways such as JNK to mediate WNT-5A-induced cell migration [67].

CD146, an adhesion molecule, can also activate RhoA and has been shown to be involved in cell migration [74]. Interestingly, WNT-5A induces redistribution of CD146 and accumulation of a unique membrane complex composed of actin, myosin IIB, and FZD_3_ (termed W-RAMP) asymmetrically at the cell periphery in a DVL- and PKC-dependent manner in melanoma cells [75]. This complex, in turn, initiates directional movement and requires RhoB- and Rab4-mediated membrane internalization and endosomal trafficking [75]. Of note, the cell movements in this context were RhoA independent. A recent study, on the other hand, has shown that WNT-5A directly binds to CD146 to activate DVL leading to activation of JNK thereby promoting formation of cell protrusions and cell migration [45]. Whether WNT-5A employs RhoA or the membrane complex-W-RAMP, for JNK activation downstream of CD146 is not clear.

Besides non-canonical WNT signaling, WNT-5A can also activate β-catenin-dependent signaling to promote cell migration. In melanoma cells, WNT-5A activates small GTPase ADP-ribosylation factor 6 (ARF6) via FZD_4_-LRP6 binding. ARF6 releases membrane-bound β-catenin from N-cadherin increasing its cytosolic abundance and triggering β-catenin-dependent transcriptional program that induces invasion and metastasis [46].

WNT-5A can also alter the adhesion properties of cells to regulate migration. For instance, WNT-5A binding to FZD_3_ activates the PI3K/AKT cascade in human dermal fibroblasts and promotes integrin-mediated adhesion of these cells [41].

Thus, WNT-5A exerts migratory effects in large number of cell and tissue types in physiological and pathological contexts.

### Stem cell differentiation and regeneration

Owing to its property of regulating cell polarity, cell movement, and cell proliferation along with the antagonistic effects on WNT/β-catenin signaling, WNT-5A may play a critical role in modulating cell fate determination and differentiation of stem cells.

Hematopoietic stem cells exhibit a shift from β-catenin-dependent to -independent WNT signaling with aging where high levels of WNT-5A are present in aged stem cells [10]. Interestingly, treatment of young hematopoietic stem cells with WNT-5A induces age-related changes such as aging-associated stem cell apolarity, reduced regenerative capacity, and an aging-like myeloid–lymphoid differentiation shift via activation of small Rho GTPase CDC42 [10]. On the other hand, reduction of WNT-5A expression in aged hematopoietic stem cells leads to their functional rejuvenation [10]. Moreover, effects of WNT-5A as observed in this study are dependent on the cell-intrinsic WNT-5A abundance and not on WNT-5A levels in stromal cells [10]. It is interesting to note that WNT-5A negatively regulates hematopoietic stem cell differentiation via inhibition of WNT/β-catenin and NFAT signaling thereby maintaining them in a quiescent stage and promoting their repopulation [73, 111, 112]. This effect is mediated by RYK-dependent inhibition of endogenous reactive oxygen species (ROS) generation [73].

Similarly, WNT-5A is also critical in mesenchymal stem cell (MSC) biology. MSCs can differentiate into multiple cell types such as adipocytes and osteocytes. Higher expression of WNT-5A is detected in MSCs as compared to committed preadipocytes which can only give rise to adipocytes [113]. Interestingly, depletion of WNT-5A in MSCs leads to their commitment to adipocytes and loss of osteocyte producing capacity demonstrating that WNT-5A is critical for the regulation of differentiation and lineage commitment of MSCs [113]. Indeed, the presence of WNT-5A in human bone marrow MSC inhibits adipogenesis and promotes osteoblastogenesis by inhibition of peroxisome proliferator-activated receptors γ (PPARγ) transactivation via a CaMKII-TAK1-TAK1-binding protein2 (TAB 2)-NLK signaling axis and simultaneous induction of runt-related transcription factor (RUNX) expression [114]. PPARγ activation is required for adipogenesis, whereas RUNX2 is critical for osteogenesis [115]. Interestingly, WNT-5A-activated PKC and ROCK signaling can also induce osteogenic differentiation in adipose-tissue-derived mesenchymal stromal cells [116]. Thus, WNT-5A functions as a master regulator determining MSC differentiation into osteogenic or adipogenic lineages.

In line with its role in morphogenesis and stem cell differentiation, WNT-5A has recently been shown to be involved in tissue repair and regeneration after injury. A study demonstrated robust induction of WNT-5A-positive mesenchymal cells following an intestinal injury which are specifically localized in the wound bed [13]. The presence of WNT-5A provided a demarcation of the regenerating proliferative area via potentiation of TGF-β signaling. This allowed a fine-tuning of regeneration and proper wound healing [13]. Increased amount of WNT-5A is observed in lung tissue from mouse model of acute respiratory distress syndrome (ARDS) which could be the repair response of damaged lungs to resolve the injury [117]. Indeed, WNT-5A can promote the survival of bone marrow derived MSCs following an oxidative-stress injury and can induce their differentiation into the type II alveolar epithelial cells via activation of JNK and PKC signaling [117].

WNT-5A also regulates spermatogenesis by supporting self-renewal and survival of spermatogonial stem cells (SSC) [9]. In contrast to hematopoietic stem cells and MSCs, SSCs do not express WNT-5A but its receptors—FZD_3_, FZD_5_, FZD_7_, and ROR2. Interestingly, WNT-5A is expressed and provided by the testicular stromal population—sertoli cells, where it promotes SSC maintenance and activity by inhibiting apoptosis in JNK-dependent manner [9].

Thus, WNT-5A may exert a highly context-dependent cell-intrinsic and -extrinsic effects in regulation of stem cell biology, regeneration, and repair.

### WNT-5A in disease

Consistent with the broad functional effects of WNT-5A during embryonic and adult life, disrupted WNT-5A signaling leads to the development of various pathological conditions in humans. We here summarize the role of WNT-5A in human pathologies such as fibrosis, inflammation, and cancer.

### Fibrosis

WNT-5A mRNA and protein expression is increased in fibroblasts obtained from lungs of usual interstitial pneumonia (UIP) patients [16]. Similarly, increased WNT-5A expression is detected in lungs following mechanical ventilation where it participates in the mechanical ventilation-induced pulmonary fibrosis [118]. WNT-5A is also present in high abundance in BAL fluid of sarcoidosis patients [119]. Augmented levels of WNT-5A are also detected in the dermal fibroblasts from keloids [120], whereas WNT-5A expression is identified in the fibrotic areas of affected human liver [121] and found increased in liver tissues from mouse model of liver fibrosis [18, 122].

Activated hepatic stellate cells (HSCs) are keys to the development of fibrotic liver by contributing the extracellular matrix (ECM) and other fibrotic factors. WNT-5A is particularly enriched in the ECM deposited by activated HSCs [121] which express more WNT-5A than the quiescent HSCs [18, 122] and normal human fibroblasts [121].

Fibroblasts from pulmonary fibrosis patients and keloid regions show increased proliferation, survival, and expression of ECM proteins [123, 124]. WNT-5A engages cAMP-PKA-CREB and PKA-GSK-3β-β-catenin pathways in dermal fibroblasts protecting them from apoptosis [79]. In line with these observations, WNT-5A promotes proliferation and survival of lung fibroblasts and also augments fibronectin and integrin expression [16]. Similarly, WNT-5A drives proliferation of and ECM deposition by activated HSCs [18]. Tissue fibrosis is an important feature of airway remodeling in obstructive lung diseases such as asthma and chronic obstructive pulmonary disease (COPD) in which airway smooth muscle can play a critical role. We have recently identified a role for WNT-5A in TGF-β-induced ECM expression in airway smooth muscle cells [21]. WNT-5A is a target of TGF-β in airway smooth muscle cells where it engages β-catenin-independent WNT signaling activating Ca^2+^-NFAT and JNK to induce ECM expression [21]. While TGF-β can regulate WNT-5A expression in airway smooth muscle cells, WNT-5A regulates expression of TGF-β in HSCs [18] underlining a critical profibrotic axis in fibrotic disorders.

In contrast of its profibrotic role, WNT-5A may be protective in diabetic renal nephropathy. High-glucose suppresses WNT-5A expression among other WNTs and promotes expression of fibrotic markers via TGF-β [125]. Forced expression or presence of recombinant WNT-5A inactivates GSK-3β thereby stabilizing β-catenin and counteracts high-glucose-induced fibrotic effects [125].

### Inflammation

WNT-5A is associated with several inflammatory disorders where it not only mediates proinflammatory cytokine and chemokine production but also regulates migration and recruitment of various immune effector cells.

Microbial pathogens [42, 126] and several proinflammatory factors such as IL-1β [31], TNF-α [30], LPS/IFNγ [15], and the IL-6 family members LIF and CT-1 [33] induce WNT-5A expression in various cell types highlighting a critical role for WNT-5A in immune responses. Abundant expression of WNT-5A is detected in the granulomatous lesions in the *Mycobacterium tuberculosis*-infected lungs [42], in the chronic periodontitis tissue [127], sera and bone marrow macrophages of patients with severe sepsis [15], the atherosclerotic lesions in humans and mouse [128], in human dental pulpitis tissues [129], in circulation and visceral fat tissues of obese patients [130], and in the synovial tissue and synovial fibrobalsts from rheumatoid arthritis patients [30, 131].

WNT-5A is associated with the maintenance of innate immune responses both in homeostasis and pathology. Basal WNT-5A expression by macrophages drives static IFN-β and -γ expression via a Rac1-NFκB pathway and also regulates expression of CD14 which is required for antigen recognition and innate immune responses during infection [132]. In addition, basal WNT-5A signaling also supports survival of macrophages as loss of WNT-5A decreases expression of prosurvival genes such as BCL-2, BCL-xl, and MCL-1, with a concomitant increase in expression of Bax, a proapoptotic protein [132]. Thus, WNT-5A is suggested to contribute to the immune system readiness for countering any future infection. Pathogenic signals such as microbes or microbial products (i.e., LPS) induce expression of WNT-5A which mediates the release of proinflammatory factors such as TNF-α, IL-6, and interferons from macrophages [132]. In addition, WNT-5A also promotes phagocytosis of microbes in a PI3K-Rac1-dependent manner. Interestingly, WNT-5A does not influence bacterial killing inside the phagosome prolonging presence of the antigen and as such might contribute to the development of sepsis by supporting sustenance of the microbial infection and persistence of proinflammatory macrophages at the site of infection [133].

WNT-5A also contributes to the immune responses by regulating the differentiation of T cells [42]. Mycobacterium infection or the presence of LPS induces WNT-5A expression in human antigen presenting cells and T cells in a TLR-NFκB-dependent manner where it mediates expression of IL-12 and IFNγ [42] contributing to the antimicrobial defense. TLR-4–MyD88 signaling is also associated with downstream effects of WNT-5A to induce expression of IL-12p40 and IL-6 in primary macrophages [134]. Similarly, LPS/IFNγ induces WNT-5A expression in macrophages where it activates CaMKII and mediates the release of IL-1β, IL-6, IL-8, and MIP1β [15].

Neutrophil recruitment to the region of infection or site of injury under the influence of various chemoattractants is another key event in innate immune response, whereas excessive neutrophilic inflammation has been linked to various diseases such as asthma and COPD. Human neutrophils express several WNT-5A receptors such as FZD_2_, FZD_5_, and FZD_8_ and treatment with WNT-5A induces the release of IL-8 and CCL2 via MAPK signaling, promoting neutrophil migration [58]. CCL2 is an important neutrophil chemoattractant and is also contributed by the macrophages. WNT-5A upregulates CCL2 expression in macrophages via JNK and NFκB signaling [135] and supernatants from LPS-treated macrophages effectively induce neutrophil migration via WNT-5A [58] emphasizing an important macrophage-neutrophil cross-talk mediated by WNT-5A.

WNT-5A has come under intense scrutiny for its role in neuroinflammatory disorders. WNT-5A induces upregulation of cyclooxygenase-2 (COX-2) expression and production of proinflammatory cytokines IL-1β, IL-6, and TNF-α in primary microglia [59]. It has also been associated with the Alzheimer’s disease-linked neuroinflammation. β-Amyloid peptide (Aβ) induces expression of WNT-5A in primary cortical neurons where it activates NFκB via upregulation of NF-κB-inducing kinase (NIK) and mediates expression of IL-1β [136]. WNT-5A-mediated Aβ-induced neuroinflammation is suggested to contribute to the neurotoxicity and Alzheimer’s disease-related neural degeneration [136].

The proinflammatory functions of WNT-5A are not only restricted to the immune cells. In human adipocytes, WNT-5A induces IL-6 and IL-1β expression [130]. In bone marrow stromal cells, LPS induces WNT-5A where it regulates expression of a plethora of proinflammatory cytokines in a MAPK- and NFκB-dependent signaling and promotes chemotactic migration of monocytes and T cell indicating a possible role in pathophysiology of rheumatoid arthritis [30]. In endothelial cells, WNT-5A augments COX-2 expression and proinflammatory cytokine production via the Ca^2+^-PKC-NFκB axis and increases vascular permeability and endothelial cell migration [137]. WNT-5A expression is induced in human dental pulp cells following TNF-α stimulation where it regulates IL-8 and CCL2 expression via a MAPK and NFκB signaling cascade and influences macrophage migration [129].

In contrast to the proinflammatory role, WNT-5A can also have opposing effect on inflammation. It has been shown to negatively regulate LPS-induced inflammatory responses in microglia by inhibiting COX-2 upregulation [138]. Another study showed that WNT-5A could function as anti-inflammatory factor by suppressing the proinflammatory M1 phenotype of macrophages in the presence of LPS/IFNγ [139] thus limiting the inflammation in various pathological situations. A dose-dependent interaction between WNT-5A and LPS could explain this discrepancy as different doses of LPS elicit differential WNT-5A responses by macrophages. It is quite plausible that low doses of LPS support proinflammatory function of WNT-5A, whereas at high LPS doses WNT-5A induces a tolerogenic phenotype in macrophages [133] thereby suppressing inflammation.

### Cancer

WNT/β-catenin signaling is closely associated with malignant disorders [140]. WNT-5A, owing to its properties of both activating and inhibiting WNT/β-catenin signaling and regulating cell movements, can be linked with cancer pathobiology. Studies have proposed both pro- and anti-tumor functions for WNT-5A and have identified several underlying signaling cascades (Table 1). Low or loss of expression of WNT-5A is linked to increased metastatic and invasive phenotype and poor prognosis in breast and colorectal cancers, whereas in thyroid cancer, it may have tumor-suppressor activity despite its increased expression [141–144]. Likewise, deletion or loss of WNT-5A expression is observed in human B cell lymphomas and myeloid leukemias [145]. On the other hand, strong expression of WNT-5A is shown in prostate cancer, acute T-cell leukemia, melanomas, and non-melanomas where it correlates with cell motility and tumor invasiveness [146–151].

**Table 1:** WNT-5A in cancer

Cancer	Expression	Signaling	Effector(s)	Consequence(s)
Prostate	Upregulated [146, 147, 168]	PKD-JNK-JunD [147] PKC-NFκB [171] Ca^2+^-CaMKII [146]	MMP1 [147, 169] BMP6 [171]	Invasion, metastasis [146, 147, 169] Proliferation [171]
Non-melanoma	Upregulated [149]	?	?	Invasion [149]
Melanoma	Upregulated [48, 148, 150]	GEP100-ARF6 [46] Ca^2+^-Calpain [167] AKT [48] Ca^2+^, CDC42 [172] PKC-STAT3 [173]	β-Catenin [46] Filamin A [167] VEGF, IL-6, MMP2 [172] LDH5 [174]	Invasion, migration [46, 150, 166, 167] EMT [165] Survival, proliferation [48] Angiogenesis [172] Immune evasion [173] Metabolic reprogramming [174]
Gastric	Upregulated [157]	FAK, Rac1 [157] PI3K-AKT [57] JNK, PKC [159]	Paxillin [157] Actin [57] Laminin γ2 [159]	Migration [57, 157–159] Tumor inflammation [160]
NSCLC	Upregulated [161, 162]	PKC-AKT [164]	BCL-2 [164]	Survival [164]
Acute ATL	Upregulated [151]	?	RANK [151]	Osteolytic lesions [151]
Colorectal^a^	Upregulated [184]	?	?	Invasion [184]
Thyroid^c^	Upregulated [143]^c^	Ca^2+^-CaMKII [143]	β-Catenin [143]^c^	(Reduced) proliferation, migration [143]^c^
Breast	Downregulated [141, 142]	CDC42 [189]	β-Catenin [188] MMP9 [189]	Tumor growth [185] Invasion [187–189]
Colorectal^b^	Downregulated [144]	?	β-Catenin [179]	Proliferation, migration [144, 179, 183]
AML/ALL	Downregulated [145]	?	?	B cell proliferation [145]
ESCC	Downregulated [156]	?	β-Catenin [156]	Proliferation, migration [156]

? unknown

^a^Early recurrence or metastatic

^b^Lymph-node negative or Dukes’ B

^c^Despite overexpression, WNT-5A is suggested to function as tumor suppressor in thyroid carcinoma, reduces β-catenin activity and proliferation and migration

Aberrant expression of components of β-catenin-independent pathway, WNT/PCP, has also been reported in Chronic lymphocytic leukemia (CLL) [152]. The study showed that WNT-5A, which is also expressed in the CLL cells, promotes polarized cell migration towards chemokine gradient (CXCL10, CXCL11, CXCL12, and CCL21) in CK1-dependent manner [152]. In another example, high expression of WNT-5A is observed in the PBMCs derived from acute T-cell leukemia/lymphoma (ATL) patients [151]. Due to its effects on osteoclast differentiation [151], WNT-5A may drive osteolytic bone lesions and hypercalcemia which are the major complications in ATL patients [153, 154].

In contrast to CLL and ATL, WNT-5A may have tumor suppressive effects in ALL. Loss of WNT-5A expression is reported in acute myeloid and acute lymphoblast leukemia [145]. WNT-5A has been shown to be epigenetically silenced by promoter hypermethylation in acute lymphoblast leukemia cells leading to the loss of expression which may drive unrestricted B cell proliferation and malignant development [155]. Indeed, WNT-5A heterozygous mice develop spontaneous B cell malignancies underlining the tumor suppressive role of WNT-5A [145].

Similarly, WNT-5A promoter hypermethylation is also observed in the esophageal squamous cell carcinoma (ESCC) tissues [156]. Ectopic expression of WNT-5A led to reduction in β-catenin signaling and inhibition of clonogenicity and motility in ESCC cell lines suggesting the tumor suppressive role of WNT-5A in ESCC [156].

WNT-5A expression is highly increased in gastric cancer and positively associates with tumor invasiveness, metastasis, and survival of the patients [157]. Administration of anti-WNT-5A antibody attenuates liver metastases of gastric cancer cells in vivo [158]. WNT-5A employs several mechanisms to regulate gastric cell invasiveness such as activation of focal adhesion kinase and Rac1 to regulate turnover of paxillin-containing adhesions [157], activation of PI3K/AKT pathway to regulate actin stress fiber formation [57], and activation of JNK and PKC signaling to induce Laminin γ2 [159] promoting cell migration. Additionally, WNT-5A abundance correlates with the expression of MCP-1 and IL-1β in gastric cancer tissues indicating that WNT-5A may drive macrophage infiltration and tumor-related inflammation [160].

WNT-5A expression is highly increased in non-small cell lung cancer (NSCLC) and has been associated with poor prognosis [161, 162]. Tobacco smoke is a very potent inducer of lung cancer [163] and exposure to cigarette smoke-extract induces WNT-5A expression in human bronchial epithelial cells [164]. WNT-5A activates PKC to upregulate anti-apoptotic genes such as BCL-2 in these cells thereby protecting them from death explaining the tumorigenic properties of WNT-5A [164].

Extensive WNT-5A expression is detected in human melanoma biopsies where it correlates with the formation of distant metastases and poor prognosis [148, 150]. WNT-5A strongly induces cell migration and invasion of melanoma cells, possibly, by inducing epithelial-to-mesenchymal transition (EMT) while decreasing the expression of metastatic suppressors [150, 165]. IL-6 induces WNT-5A in melanoma cells via p38 which, in turn, mediates cell migration [166]. As discussed earlier, WNT-5A activates ARF6 in melanoma cells leading to disruption of N-cadherin-β-catenin interaction, enhanced β-catenin-mediated transcription and invasion [46]. It can also activate Ca^2+^-dependent signaling leading to the activation of calpain protease which cleaves filamin A. Cleavage of filamin A induces cytoskeletal remodeling and cell motility [167]. WNT-5A can also confer a survival advantage to melanoma cells, thereby negatively influencing the outcome of therapeutic approaches. Prolonged treatment with BRAF inhibitors induces WNT-5A expression in melanoma cells and contributes to the development of resistance to BRAF inhibitor-induced apoptosis [48]. This process involves FZD_7_- and RYK-mediated activation of prosurvival AKT signaling [48]. Knock-down of endogenous WNT-5A decreases melanoma cell proliferation and sensitizes them to BRAF inhibitor-induced cell death [48].

WNT-5A regulates motility in prostate cancer cells as well by promoting actin remodeling via Ca^2+^-CaMKII signaling [146]. Prostate cancer tissues show increased expression of WNT-5A [146, 168] promoting migration and invasiveness [147]. WNT-5A signaling through ROR2 and FZD_2_ activates protein kinase D (PKD) and JNK to induce Matrixmetalloprotease 1 (MMP1) expression via JunD [147]. MMP1 expression is important for prostate cancer cell invasiveness and bone metastasis [169]. Bone is a major site for metastasis of various tumors including prostate cancer. Prostate cancer cells show increased migration towards bone marrow stromal cells which is suppressed in the presence of WNT-5A siRNA-transfected bone marrow stromal cells, suggesting that WNT-5A can also function as a chemoattractant or homing factor for prostate cancer cells [170]. The prostate cancer and bone cross-talk also promotes prostate cancer cell proliferation. WNT-5A expressed by bone marrow stromal cells induces expression of BMP-6 in prostate cancer cells via a PKC-NFκB pathway [171]. BMP-6, in turn, activates SMAD and β-catenin signaling to promote proliferation in prostate cancer cells [171]. Indeed, considerable nuclear β-catenin staining is found in prostate cancer tissues [147]. This signaling mechanism also explains the development of castration-resistant prostate cancer phenotype. Prostate cancer cells require androgens for their growth and as such androgen restriction is first-line therapy for prostate cancer patients. With time, considerable subsets of patients develop androgen-resistant prostate cancer. WNT-5A induced BMP-6, thus contributes to the proliferation of prostate cancer cells in the absence of androgens [171].

Studies have suggested a broader function for WNT-5A in cancer than just cell growth and invasion. For instance, it can relay immunomodulatory and proangiogenic functions or modulate cell survival. WNT-5A induces the release of IL-6, MMP2, and vascular endothelial growth factor (VEGF) containing exosomes from melanoma cells in a Ca^2+^- and CDC42-dependent process that requires cytoskeletal reorganization [172]. Co-culture of WNT-5A-deficient melanoma cells with endothelial cells suppresses endothelial cell branching, whereas treatment of endothelial cells with exosomes isolated from WNT-5A-treated melanoma cells induces angiogenesis highlighting a proangiogenic role for WNT-5A [172]. WNT-5A also suppresses expression of tumor-associated antigens in melanoma cells via activation of PKC and STAT3. This leads to impaired cytotoxic T-cell clearance of tumor cells [173]. Interestingly, WNT-5A can drive metabolic reprogramming in cancer cells by inducing lactate dehydrogenase 5 (LDH5) leading to an increase in anaerobic glycolysis [174]. The serum level of LDH is an important predictor of prognosis and treatment response in melanoma patients [175]. WNT-5A and LDH5 expression levels positively correlate in melanoma patient tissue samples [174]. This is particularly important as strong staining of both WNT5A and LDH5 is linked with reduced disease-free survival in melanoma patients [148, 174]. Contrary to its effects in melanoma cells, WNT-5A increases oxidative phosphorylation rates in breast cancer cells demonstrating a context-dependent function of WNT-5A that can also explain its tumor-promoter and tumor-suppressor roles [174].

What drives increased expression of WNT-5A in cancer cells? A study has found that microRNA-26a expression is reduced in prostate cancer cells [176]. miR-26a suppresses WNT-5A and forced expression of miR-26a attenuates cell proliferation, metastasis, and EMT, and induced G1 phase arrest suppressing WNT-5A expression and inhibiting prostate cancer progression [176]. Epigenetic mechanisms could also participate in the aberrant expression of WNT-5A in cancer cells. Hypomethylation of the WNT-5A promoter in prostate cancer cells accounts for the increased transcription of WNT-5A in these cells [177]. In another scenario, reduced expression of WNT-5A antagonists such as Klotho might contribute to increased availability and signaling of WNT-5A in cancer cells [178]. Expressions of Klotho and WNT-5A are inversely correlated in melanoma tissues, whereas the presence of Klotho suppressed melanoma cell invasion [178].

In addition to tumor-promoting activity, WNT-5A also functions as tumor suppressor in few cancer types. In colorectal cancer (CRC), loss of WNT-5A is frequently observed and associated with poor prognosis and survival [144]. In line with this, methylation of the WNT-5A promoter is observed in metastatic CRC cell lines explaining low abundance of WNT-5A in CRC [179, 180]. Promoter methylation of WNT-5A is associated with distinct tumor subtypes in colorectal cancer [181, 182]. Treatment of CRC cells with Genistein, a soy flavonone and tyrosine kinase inhibitor with protective activity in CRC, reduces WNT-5A promoter methylation thereby increasing WNT-5A gene expression and inhibiting cell proliferation [183]. WNT-5A also attenuates migration of colon cancer cell lines [144]. As activated WNT/β-catenin is associated with CRC, ectopic expression of WNT5A resulted in substantial inhibition of tumor cell clonogenicity of CRC cells, with downregulation of intracellular β-catenin protein level and concomitant decrease in β-catenin activity [179].

In a contrasting study, increased WNT-5A expression is associated with poor prognosis in CRC patients and WNT-5A promoted directional cell migration and invasion in CRC cells. However, increased expression of WNT-5A is not sufficient to augment malignancy or metastasis in APC-driven intestinal tumor model [184] suggesting that additional, not yet understood, mechanisms govern WNT-5A activity at different stages of cancer pathogenesis. While further studies are required to elucidate a clear role of WNT-5A in CRC, it is tempting to speculate that WNT-5A acts as a tumor suppressor in β-catenin-dependent stages of CRC progression.

Loss of WNT-5A is observed in primary invasive breast cancers and is associated with higher histological grade and rapid appearance of distant metastases leading to shorter recurrence-free survival in these patients [141, 142]. The low abundance of WNT-5A in breast cancer cells could be attributed to epigenetic silencing of the WNT-5A promoter. Elevated expression of protein inhibitor of activated STAT 1 (PIAS1) is found in breast cancer tissues and it has been shown to associate with methylated regions of WNT-5A promoter in breast cancer cells [185]. PIAS1, a transcriptional regulator, is known to recruit DNA methyltransferases (DNMTs) thereby regulating promoter methylation. Of note, knock-down of PIAS1 coincides with reduced methylation and increased acetylation of the WNT-5A promoter indicating gene activation with a subsequent increase in WNT-5A expression. It leads to reduction in the number of tumor-initiating cells and attenuates breast cancer growth in vivo suggesting that epigenetic silencing of WNT-5A via PIAS1 is an important feature in breast cancer [185]. Additionally, the low WNT-5A expression could also be due to posttranslational suppression of WNT-5A mRNA in breast cancer cells by HuR proteins. Of note, HuR expression is highly augmented in invasive breast cancer cells [36]. Further, miRNA-374a is highly increased in breast cancer tissues and is associated with poor metastasis-free survival [186]. miRNA-374a promotes EMT and metastasis in breast cancer cells both in vivo and in vitro via targeted downregulation of negative regulators of WNT/β-catenin signaling such as WNT-5A [186].

The tumor suppressive function could also be attributed to adhesion promoting function of WNT-5A in certain cell types. WNT-5A could regulate mammary epithelial cell adhesion by phosphorylating Discoidin domain receptor 1 and activating its interaction with collagen thereby negatively regulating cell migration [187]. Similarly, WNT-5A stimulation of breast epithelial cells increases adhesion by inducing CK1α-dependent phosphorylation of β-catenin which, in turn, promotes E-cadherin-β-catenin association [188]. This stabilizes adheres junctions and attenuates β-catenin transcriptional function [188]. WNT-5A activates CDC42 in various cell types including breast cancer cells. A study found that WNT-5A-activated CDC42 limits ERK1/2 activation and subsequent MMP9 expression. This is suggested to restrain cell migration and invasiveness in breast cancer [189]. In agreement with these observations, small WNT-5A-derived peptides could increase adhesion and decrease metastasis and invasion of breast cancer cells both in vitro and in vivo [190, 191].

In contrast to the tumor-suppressor function of WNT-5A in breast cancer, studies have also suggested a cell migration-promoting role for WNT-5A. In a breast cancer cell line MDA-MB-231 which expresses very low endogenous WNT-5A, stimulation with WNT-5A activated a DVL2- and Daam1-dependent RhoA signaling inducing cell migration [110], whereas in another breast cancer cell line with high endogenous WNT-5A levels (MCF-7), WNT-5A can promote cell migration via a DVL2-Rab35-Rac1-dependent and RhoA-independent signaling [109]. In a contrasting study using MCF-7 cell line, WNT-5A attenuated filopodia formation and cell migration via activation of cAMP-regulated phosphoprotein of 32 kDa (DARPP-32) and CREB [192]. Interestingly, macrophages associated with primary breast cancer tissues have been shown to express WNT-5A [193]. The co-culture of MCF-7 with macrophages promotes WNT-5A expression in macrophages and invasiveness of MCF-7, a feature which was also recapitulated by direct stimulation of MCF-7 with recombinant WNT-5A [193]. Similarly, microglia, the resident brain macrophages, have been shown to enhance breast cancer cell (MCF-7) invasion in a WNT-dependent manner [194]. The study showed microglia transporting breast cancer cells into the brain tissue [194]. Of note, WNT-5A has been shown to induce proliferation and invasion of microglia [59]. While the pro-cell migratory effects of WNT-5A in breast cancer require further studies, it is quite possible that WNT-5A regulates breast cancer metastasis depending on the tumor-microenvironment communication.

The opposing roles for WNT-5A in cancer are intriguing and are matter of intense investigation. As WNT-5A antagonizes WNT/β-catenin signaling, it is tempting to speculate that it functions as tumor suppressor in WNT/β-catenin-dependent cancers provided it activates the downstream cascade involved in this antagonism. The pro-tumor activity might be attributed to the cell migratory, proliferative. and prosurvival effects of WNT-5A. Moreover, the differential role of WNT-5A could also be due to different properties of recently characterized WNT-5A isoforms. WNT-5A promoter generates two identical transcripts utilizing alternative transcription start sites—WNT-5A-L and WNT-5A-S [24–26]. While WNT-5AL inhibits tumor growth, WNT-5AS promotes it. Expression of these two isoforms is altered in breast cancer, cervix carcinoma, and aggressive neuroblastomas where WNT-5A-L is downregulated and WNT-5A-S is most abundantly expressed [25]. Thus, not only the downstream signaling but also the abundance of specific isoforms can contribute to the differential effects of WNT-5A in cancer. Thus, the downstream effects of WNT-5A are highly context dependent and the differential signaling mechanisms it engages may account for the opposing functions of WNT-5A in cancer.

### WNT-5A as a therapeutic target

While we still await a clear understanding of WNT-5A biology, development of certain WNT-5A mimicking molecules and their beneficial effects in animal models of diseases raise hopes for therapeutic targeting of WNT-5A for curing deadly diseases. Foxy5 is a WNT5A derived N-formylated hexapeptide which mimics tumor suppressive effects of WNT5A on breast cancer both in vitro [190, 191] and in vivo [191]. The presence of Foxy5 has anti-migratory effects on breast cancer cell line [190, 191] and administration of Foxy5 has been shown to prevent lung and liver metastases in a mouse model of breast cancer [191]. The substitution of N-terminal formyl group of Foxy5 with a t-butoxycarbonyl group (t-boc) reversed its function turning Foxy5 into WNT-5A antagonist, termed Box5 [195]. Box5 antagonizes WNT-5A-induced melanoma cell invasion [195] and prevents β-amyloid peptide-induced WNT-5A-dependent inflammation and neurotoxicity in mouse cortical cultures [136].

Likewise, UM206, a oligopeptide with high homology to WNT-5A, functions as a FZD_1_/FZD_2_ antagonist with therapeutic benefit in reducing cardiac remodeling an animal model of myocardial infarction [196]. Although the effects of UM206 cannot be attributed specifically to WNT-5A as the peptide also blocks signaling induced by WNT-3A, WNT-5A is known to regulate fibroblast proliferation, migration, and activation leading to matrix remodeling [16, 103].

## Conclusion

WNT-5A is a pleotropic growth factor with wide-ranging effects in different cells and tissues, regulating key functions throughout the human life span. While it is indispensable for proper embryonic development, it is equally critical for maintenance of tissue homeostasis in adult life. Simultaneously, derailed WNT-5A signaling results in various pathological disorders in humans. Understanding the mechanisms involved in the maintenance of WNT-5A homeostasis such as its inducers and signaling partners, both positive and negative modulators, is key for therapeutic targeting of this important WNT in various diseases.
